# A new compass for health psychology

**DOI:** 10.3389/fpsyg.2013.00724

**Published:** 2013-10-09

**Authors:** Francesco Pagnini

**Affiliations:** ^1^Department of Psychology, Catholic University of MilanMilan, Italy; ^2^Niguarda Ca' Granda HospitalMilan, Italy

**Keywords:** health psychology, clinical psychology, mind-body relations, mind-body therapies, quality of life, book review, health

The term “Health Psychology” refers to the application of psychological knowledge and methods to health, illness, and healthcare. The final aim of the discipline is the promotion and the improvement of the well-being as a whole, focusing on the psychological aspects. Health psychologists investigate the mind/body relationship, as well as mental issues related to medical conditions. They provide information about these topics and promote prevention and interventions.

The handbook edited by Irving Weiner and colleagues is about health, with a primary focus on the psychological aspects. The bio-psycho-social approach adopted reflects the complexity of the human health, that includes in its etymology the concept of “whole.” As pointed out in the first chapter by David Marks, there are many elements that interact within the concept of health, including physical, psychosocial, cultural, economical, and spiritual aspects. People who take care of others' health should be aware of this complexity. The book does not forget to remind it, providing a multifaceted and multidimensional view on each treated topic.

The reader will find a comprehensive description of the state-of-the-art in many topics of health psychology, including stress, coping and adaptation to the disease, intervention and prevention strategies. It is valuable that historical perspectives and research trends are presented. Many questions that arise during the reading will find an answer in the text. For example, while reading about a certain medical condition, the reader may be curious about the application of a certain psychological theory or technique. However, some questions remain opened, indicating a need of further studies and future research trends.

Treated topics cover an overview of conceptual and professional issues, an in-depth analysis of great number of medical diseases, attention to health psychology changes across some developmental stages, such as the life span, and contextual issues, such as gender, culture, and ethnicity. Moreover, primary care psychology, together with complementary and alternative therapies are analyzed. Considered medical diseases include asthma, obesity, smoking, musculoskeletal conditions, diabetes, HIV/AIDS, headaches, cancer, pain, insomnia, coronary heart disease and hypertension, gastrointestinal diseases, and spinal cord injury.

Potential readership refers to all people who are interested in health psychology topics, including grad students, researchers interested in having up-to-date information, clinical psychologists that work with medical patients, other clinical professionals, such as physicians and nurses. Students will find a comprehensive introduction to health psychology topics, while professionals will find a source of update of their knowledge. Moreover, the reading of this handbook can also be interesting from a naïve perspective. Health is a puzzle that everybody wants to solve for their own. A puzzle that needs many pieces to thrive.

